# Extract of *Cynomorium songaricum* ameliorates mitochondrial ultrastructure impairments and dysfunction in two different in vitro models of Alzheimer’s disease

**DOI:** 10.1186/s12906-021-03375-2

**Published:** 2021-08-09

**Authors:** Dan Cheng, Lei Su, Xu Wang, Xinjie Li, Lingling Li, Mengyuan Hu, Yi Lu

**Affiliations:** 1 School of Chinese Medicine, Beijing University of Chinese Medicine, Beijing, China; 2 School of Chinese Medicine, LKS Faculty of Medicine, The University of Hong Kong, Hong Kong, China

## Abstract

**Background:**

Alzheimer’s disease (AD) is one of the most common neurodegenerative disorders, but there is still no effective way to stop or slow its progression. Our previous studies demonstrated that extract of *Cynomorium songaricum* (ECS), a Chinese herbal medicine, had neuroprotective effects in AD models in vivo. However, the pharmacological mechanism of ECS in AD is still unclear.

**Methods:**

To study the mechanisms of action of the effects of ECS on AD, we used Aβ_25–35-_ and H_2_O_2_-exposed HT22 cells to mimic specific stages of AD in vitro. The mitochondrial membrane potential (MMP), intracellular ATP, intracellular reactive oxygen species (ROS), and expression levels of mitochondrial dynamics-related proteins in each group were examined. Furthermore, we explored the mechanisms by which ECS reduces the phosphorylation of Drp1 at Ser637 and the changes in the concentrations of intracellular calcium ions in the two models after FK506 intervention.

**Results:**

The results showed that ECS significantly enhanced the MMP (*P* < 0.05), increased intracellular ATP levels (P < 0.05) and decreased intracellular ROS levels in the Aβ- and H_2_O_2_-induced cell models (*P* < 0.05). Additionally, ECS regulated the expression levels of mitochondrial dynamics-related proteins by reducing the phosphorylation of Drp1 at Ser637 (*P* < 0.05) and decreasing the expression of Fis1 in the H_2_O_2_-induced models (*P* < 0.05). Further study indicated that ECS reduced the overload of intracellular calcium (*P* < 0.05).

**Conclusion:**

Our study results suggest that ECS protects the mitochondrial ultrastructure, ameliorates mitochondrial dysfunction, and maintains mitochondrial dynamics in AD models.

**Supplementary Information:**

The online version contains supplementary material available at 10.1186/s12906-021-03375-2.

## Background

Alzheimer’s disease (AD) is one of the most common neurodegenerative disorders in elderly individuals, and it can cause cognitive dysfunction and interfere with the quality of life of patients. The two pathological characteristics of AD are intracellular neurofibrillary tangles (NFTs) and extracellular senile plaques (SPs) in the hippocampus [1]. NFTs are formed by the misfolding of highly phosphorylated Tau protein, while SPs are formed mainly from the deposition of amyloid-beta (Aβ) peptides [1]. These misfolded protein aggregates have high neurotoxicity and synaptotoxicity, leading to a cascade of neurodegeneration during the progression of the disease [2].

The deposition of Aβ may result from the continuous interactions of other pathophysiological mechanisms [3]. Therefore, some researchers have argued that the hypothesis suggesting that Aβ directly causes AD may not be correct [4]. They have proposed that focusing on the early stage of AD and implementing effective interventions are promising new treatment strategies [5]. Mitochondrial dysfunction occurs at the early stage of AD, and it is considered to be an intracellular process that is severely compromised in AD [5–8]. On the other hand, mutations in β-amyloid precursor protein (APP) generate Aβ [9], which leads to the accumulation of Aβ on the mitochondrial membrane and ultimately causes mitochondrial dysfunction [10–13]. Mitochondrial dysfunction affects the production of reactive oxygen species (ROS) [14]; moreover, the molecular targets regulated by ROS, including mitochondrial DNA (mtDNA), calcium homeostasis, mitochondrial dynamics and function, and energy homeostasis, are affected by mitochondrial dysfunction [15–17]. Excessive ROS production exacerbates mitochondrial dysfunction, disrupts intracellular calcium balance, opens mitochondrial permeability transition pores (MPTPs), and leads to a decrease in mitochondrial membrane potential (MMP), thereby contributing to a vicious cycle [17, 18].

The term “mitochondrial dynamics” refers to fission and fusion in mitochondria, which play critical roles in mitochondrial dysfunction and the pathogenesis of AD [15, 19]. An imbalance in mitochondrial dynamics significantly changes the expression levels of mitochondrial dynamics-related proteins, including dynamin-related protein 1 (Drp1), fission 1 (Fis1), mitofusin l (Mfn1), mitofusin 2 (Mfn2), and optic atrophy 1 (Opa 1) [20]. In addition, phosphorylation of Drp1 promotes mitochondrial division and leads to abnormal mitochondrial dynamics [21]. Therefore, it is possible to protect mitochondrial function by inhibiting the expression of p-Drp1 in mitochondria [22].

Some natural products, such as coenzyme Q10 and curcumin, have been reported to reduce Aβ deposition in mitochondria, improve mitochondrial dysfunction, restore neuroplasticity, and improve cognitive dysfunction in patients [23]. Some Chinese medicine extracts have also been studied in this field, and they have shown promising effects for breakthrough treatments. *Cynomorium songaricum Rupr* (*C. songaricum*), a traditional Chinese medicine, is widely used to treat sexual disorders, such as erectile dysfunction and menstrual problems [24, 25]. Our previous studies have shown that extract of *C. songaricum* (ECS) has neuroprotective effects in vivo and in vitro. It can alleviate the behavioral changes and morphological damage in animals with AD and resist the cytotoxicity induced by Aβ in vitro [26–29]. Additionally, by ultrahigh-performance liquid chromatography and mass spectrometry (UPLC/LTQ-Orbitrap MS), we have identified that the main chemical components of ECS are B-type oligomeric forms of flavonoids [30]. However, the pharmacological effects and mechanisms of action of ECS on AD are not entirely clear. This study will identify whether ECS ameliorates AD in vitro by regulating mitochondrial dynamics homeostasis and maintaining the mitochondrial function of HT22 cells treated with Aβ and H_2_O_2_.

## Materials and methods

### Standard solution and sample extraction

The reference compounds phlorizin, isoquercitrin, and epicatechin (Yuanye Bio-Technology Corporation, Shanghai, China) were dissolved in 70% methanol (Fisher Corporation, Shanghai, China) and then stored at 4 °C for susequent qualitative analysis.

We obtained prepared slices of *Cynomorium songaricum* from the Chinese Medicine Clinic of Beijing University of Chinese Medicine. The extraction method of ECS was optimized based on our previous study [26]. Briefly, the plants were ground into a powder and sieved through 60 mesh, and then 500 g of the dried plant powder was extracted with 800 mL of methanol via ultrasound-assisted extraction (KQ 3200 dB, 100 W, 40 kHz, Kunshan, China) three times for 60 min each time. The three extracts were filtered and combined, and then the combined filtrate was evaporated in a rotary evaporator (RE 301, Shanghai, China) at 40 °C to obtain a dry extract of methanol. Next, the dry extract was dissolved in 300 mL of water and successively extracted with dichloromethane and ethyl acetate three times to obtain ECS. The ECS was evaporated at 30 °C, and its dried extract was dissolved in 70% methanol and stored at 4 °C for quality analysis or dissolved in PBS for pharmacological study.

### HPLC analysis

#### Apparatus and chromatographic conditions

The qualitative analysis was performed on a 1100 series High-Performance Liquid Chromatography (HPLC) system (Agilent, California, America). All the samples were analyzed at a column temperature of 30 °C with a Kromasil C18 column (4.6 mm × 250 mm, 5 μm, Akzo Nobel, Sweden). The mobile phase consisted of eluents A (acetonitrile) and B (0.1% formic acid solution), and the optimized gradient elution model was as follows: 0–20 min, 10–18% A; 20–30 min, 18–22% A; 30–45 min, 22–40% A; 40–55 min, 40–70% A. The flow rate was 0.8 mL/min, and 10 μL of the sample was injected into the HPLC instrument each time. The detector scanned from 190 nm to 400 nm, and the optimal detection wavelength was 280 nm.

#### Validation of the method

According to ICH guidelines, the analytical method was determined with suitable levels of sample linearity, precision, and accuracy [31]. All calibration curves were established by using six different concentrations of mixed standards.

### Cell culture and treatment

HT22 mouse hippocampal neuronal cells were cultured in Dulbecco’s modified Eagle’s medium (DMEM, Gibco, USA) supplemented with 10% fetal bovine serum (FBS, Gibco) and 1% penicillin-streptomycin solution (Invitrogen, USA) at 37 °C in a 5% CO_2_ incubator.

HT22 cells were pretreated with ECS for 2 h, and then Aβ_25–35_ or hydrogen peroxide (H_2_O_2_) was added to the final concentration (see below). Aβ_25–35_ plaques were generated as described in a previous method [32]. Briefly, Aβ_25–35_ monomers were dissolved in distilled water to a stock concentration of 1 mmol/L, incubated at 37 °C for 7 days, and then stored as Aβ_25–35_ plaques at − 20 °C for later use.

### Cell viability assay

HT22 cells were seeded in 96-well plates at a density of 5 × 10^3^ cells/well and pretreated with a series of concentrations of ECS from 20 to 800 μg/mL for 2 h. Then, certain concentrations of Aβ_25–35_ and H_2_O_2_ were added and cocultured with ECS for another 22 h. Cell viability in response to the different treatments was measured using a cell counting kit-8 (CCK-8) (Dojindo, Japan). Briefly, after each treatment, 10 μL of CCK-8 solution was added to each well and incubated for 2 h. The absorbance in each well was read at 450 nm on a microplate reader (Thermo Fisher Scientific, USA). Cell viability was determined using the following equation: Cell viability (%) = (Mean _OD drug-treated cells_)/(Mean OD _untreated cells_) × 100%.

### Transmission electron microscopy (TEM)

After ECS treatment, cells were collected with trypsinization, washed three times with phosphate-buffered saline (PBS, Corning, USA), and then fixed in a 2.5% glutaraldehyde solution at 4 °C overnight. The glutaraldehyde solution was discarded, and the cells were washed three times with PBS. The cells were postfixed with 1% osmium tetroxide for 3 h, stained with 2% uranyl acetate at 4 °C, dehydrated in a graded ethanol series with concentrations ranging from 50 to 100%, and embedded in Spurr resin. Ultrathin (60 nm) sections were stained with 3% uranyl acetate and lead citrate and then examined by TEM (JEM-1400Plus).

### Flow cytometric analysis

We applied an oxidation-sensitive fluorescent probe, 2′,7′-dichlorofluorescein diacetate (DCFH-DA), from an assay kit (Beyotime Biotechnology, Beijing, China) to label intracellular ROS and detected the 2′,7′-dichlorofluorescein (DCF) intensity by flow cytometry. Briefly, HT22 cells were seeded into six-well plates at a density of 2 × 10^5^ cells/well and treated as described above. After treatment, the cells were washed twice in PBS, collected, adjusted to 1× 10^6^ cells/mL, and incubated with DCFH-DA at 37 °C for 20 min. DCFH-DA was intracellularly deacetylated by a nonspecific esterase, which was further oxidized by ROS to produce the fluorescent compound 2,7-dichlorofluorescein (DCF). The fluorescence intensity of DCF was detected by flow cytometry (BD FACSCalibur, USA), and the stained cells emitting fluorescence were measured using channel FL-1. The results were measured as the relative mean fluorescence intensity of FL-1 of the control group [33].

### Western blot analysis

After treatment, HT22 cells were lysed in radioimmunoprecipitation assay (RIPA) buffer (Sigma-Aldrich, USA) supplemented with a protease inhibitor cocktail (Roche Diagnostics, Shanghai, China). Total protein quantification and western blot procedures were performed routinely [34]. Then, equal amounts of proteins were resolved by sodium dodecyl sulfate-polyacrylamide gel electrophoresis (SDS-PAGE) and transferred onto polyvinylidene difluoride (PVDF) membranes. The membranes were incubated with blocking buffer, 5% nonfat milk, or 5% BSA (for phosphoproteins) for 1.5 h. Then, the membranes were incubated with primary antibodies against Drp-1, Mfn1, Mfn2, Fis1, β-actin (Proteintech, Wuhan, China), p-Drp1 (Ser637), and Opa1 (Cell Signaling Technology, CST, USA). After that, the membranes were incubated with corresponding secondary antibodies, namely, goat anti-rabbit IgG or goat anti-mouse IgG (Proteintech, Wuhan, China). The proteins were detected using an enhanced chemiluminescence (ECL) kit (Thermo Fisher Scientific, USA) and visualized by a chemiluminescence imaging system (Azure 300, Azure Biosystems, USA).

### Fluorospectrophotometry

The intracellular ATP level was measured using a luminescent ATP detection assay kit (Beyotime, Beijing, China) according to the kit protocol. Briefly, the medium was discarded, and the cells were washed three times with PBS. Then, 1% Triton X-100 (Sigma, USA) was added to lysed cells, and the cells were incubated for 30 min. Finally, 90 μL of ATP assay kit reagent was added to 10 μL of cell lysate in each well of the 96-well plates, and luminescence was measured by a fluorospectrophotometer.

### Fluorescence microscopy analysis

Fluo-4 acetoxymethyl ester (Fluo-4-AM) (Beyotime, Beijing, China) dye was used to measure intracellular calcium. HT22 cells were seeded into 6-well plates at a density of 2× 10^5^ cells/well and treated as described above. After treatment, the cells were stained with Fluo-4-AM diluted to a concentration of 2.5 μM for 30 min in darkness at 37 °C and then washed with Dulbecco’s phosphate-buffered saline (DPBS) three times. The green fluorescence, which reflected the intracellular calcium level, was recorded by a fluorescence microscope (Olympus, Japan).

An MMP assay kit with JC-1 (Beyotime, Beijing, China) was used. The kit resulted in the formation of JC-1 aggregates that emitted red fluorescence at a high MMP, while monomers that emitted green fluorescence at low MMP were produced when they selectively entered the mitochondria. After treatment as described, the cells in different groups were washed with PBS twice and then incubated with 2 μM JC-1 in fresh culture medium under permissive conditions. After 30 min, the cells were washed with PBS three times and resuspended in culture medium. Red and green fluorescence was detected by a fluorescence microscope (Olympus, Japan).

### Statistical analysis

The statistical analyses were performed using SPSS 22.0 software (SPSS Inc., Chicago, USA). All data from the cell experiments are presented as the mean ± SE. The statistical significance was analyzed with one-way analysis of variance (ANOVA) followed by Tukey’s HSD post hoc test, and *P* < 0.05 was considered to indicate statistical significance.

## Results

### Validation of the quantitative analysis method

**Fig. 1:**
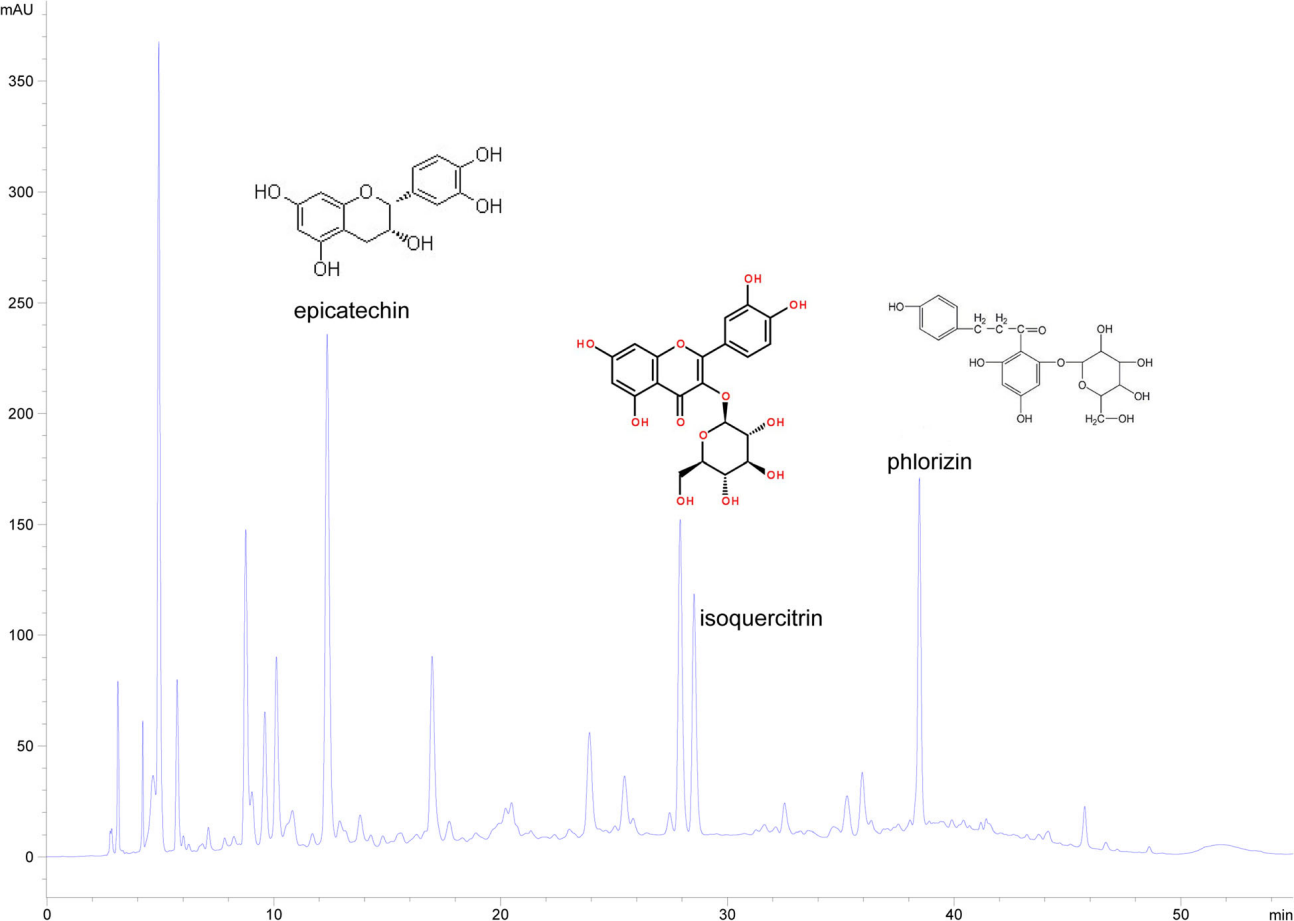
Chemical structures of the standard compounds and the chromatogram of ECS. Quantitative analysis of ECS was performed by HPLC, and the three compounds, epicatechin, isoquercitrin, and phlorizin, were labeled in the chromatogram of ECS based on the relative retention times of their standards

The quantitative method involved determination of linearity, the LOD, the LOQ, precision, reproducibility, stability, and recovery. Three standards, epicatechin, phlorizin, and isoquercitrin, were used to obtain reference peaks to validate the method (Fig. 1). Standard curves and linearity were generated by plotting the peak areas (y) against the compound concentrations (x, μg mL^− 1^), which were obtained as shown in Table 1. There was good linearity within a wide concentration range. The LOD (signal/noise = 3) and LOQ (signal/noise = 10) values of the three compounds were also calculated and found to be within the ranges of 0.0317–0.0655 μg/mL and 0.0944–0.1567 μg/mL, respectively.

**Table 1:** Linearity, the limits of detection (LOD), and the limits of quantification (LOQ) for the three compounds

Compounds	Calibration curve^a^	R^b^	Linear range (μg/mL)	LOD (μg/mL)	LOQ (μg/mL)
epicatechin	y = 652.34x + 3.0036y	0.9999	4.50–144	0.0427	0.0944
phlorizin	y = 1858.9x - 48.333	1.0000	3.68–117.6	0.0655	0.7704
isoquercitrin	y = 1304.4X-10.637	0.9998	2.77–177.3	0.0317	0.1567

^a^ y, peak area; x, compounds concentration (μg/mL); ^b^ R, correlation coefficient, *n* = 6

Six repeated injections of the same sample were used to evaluate repeatability, and gradient dilutions of six concentrations of the standard compounds was used to validate their precision. The relative standard deviations (RSDs) of the reference peaks were 0.33–0.39% and 0.65–1.12%, respectively. The stability of the sample solutions was evaluated by injecting the same sample at 0, 2, 4, 8, 12, and 24 h. The RSD of stability was less than 1%, indicating that the solution remained stable within 24 h. The recovery of the three compounds was evaluated by adding the same amounts of the standards to a certain amount of sample. As shown in Table 2, the recovery of the three compounds was 98–103%, and the RSDs of recovery ranged from 0.54 to 1.1%. These results suggested that the optimized method was suitable for the following quantitative and qualitative analyses.

**Table 2:** Precisions, Repeatability, Stability and Recovery of the three standard compounds

Compounds	Precisions RSD (%) (*n* = 6)	Repeatability RSD (%) (*n* = 6)	Stability RSD (%) (*n* = 6)	Recovery (%) (*n* = 9)	RSD (%)
epicatechin	0.33	1.12	0.33	98.54	1.1
phlorizin	0.39	0.65	0.38	102.47	0.54
isoquercitrin	0.21	1.31	0.22	102.71	0.89

### Effects of ECS on HT22 cell viability in two models

The effects of different concentrations and administration times of ECS on the viability of HT22 cells were examined, and the optimal administration concentration of ECS was determined to be 200 μg/mL, while the optimal action time was 24 h (supplement file, Fig. S1). Furthermore, 20 μM Aβ and 200 μM H_2_O_2_ were used to mimic the AD model in vitro (supplement file**,** Fig. S2, Fig. S3).

**Fig. 2:**
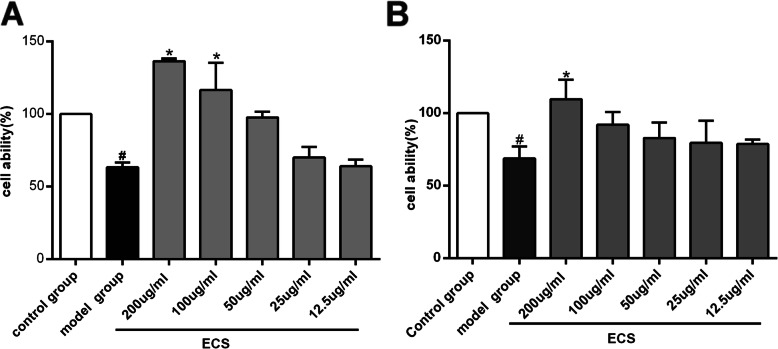
Effects of ECS on viability in the Aβ-induced model and the H_2_O_2_-induced model. **A** Effects of different concentrations of ECS on the viability of Aβ-exposed cells after 24 h. **B** Effects of different concentrations of ECS on the viability of H_2_O_2_-exposed cells after 24 h. All values are shown as the means ± SEMs from three independent studies. Statistical analyses were performed using one-way ANOVA and Tukey’s HSD post hoc comparisons. ^#^*p* < 0.05, vs. control group; * *p* < 0.05, vs. model group

As shown in Fig. 2, 100 μg/mL and 200 μg/mL ECS significantly improved the cell viability of the Aβ-exposed cells (*P < 0.05), which exhibited cell viabilities of 116.63 ± 18.65% and 136.48 ± 1.40%, respectively. Therefore, 200 μg/mL ECS was used to treat HT22 cells induced by Aβ in subsequent experiments. In the H_2_O_2_-induced model, 200 μg/mL ECS significantly improved cell viability (102.19 ± 14.10%, *P < 0.05). Thus, 200 μg/mL ECS was determined to be effective in H_2_O_2_-exposed cells.

### Beneficial effects of ECS on mitochondrial ultrastructures

**Fig. 3:**
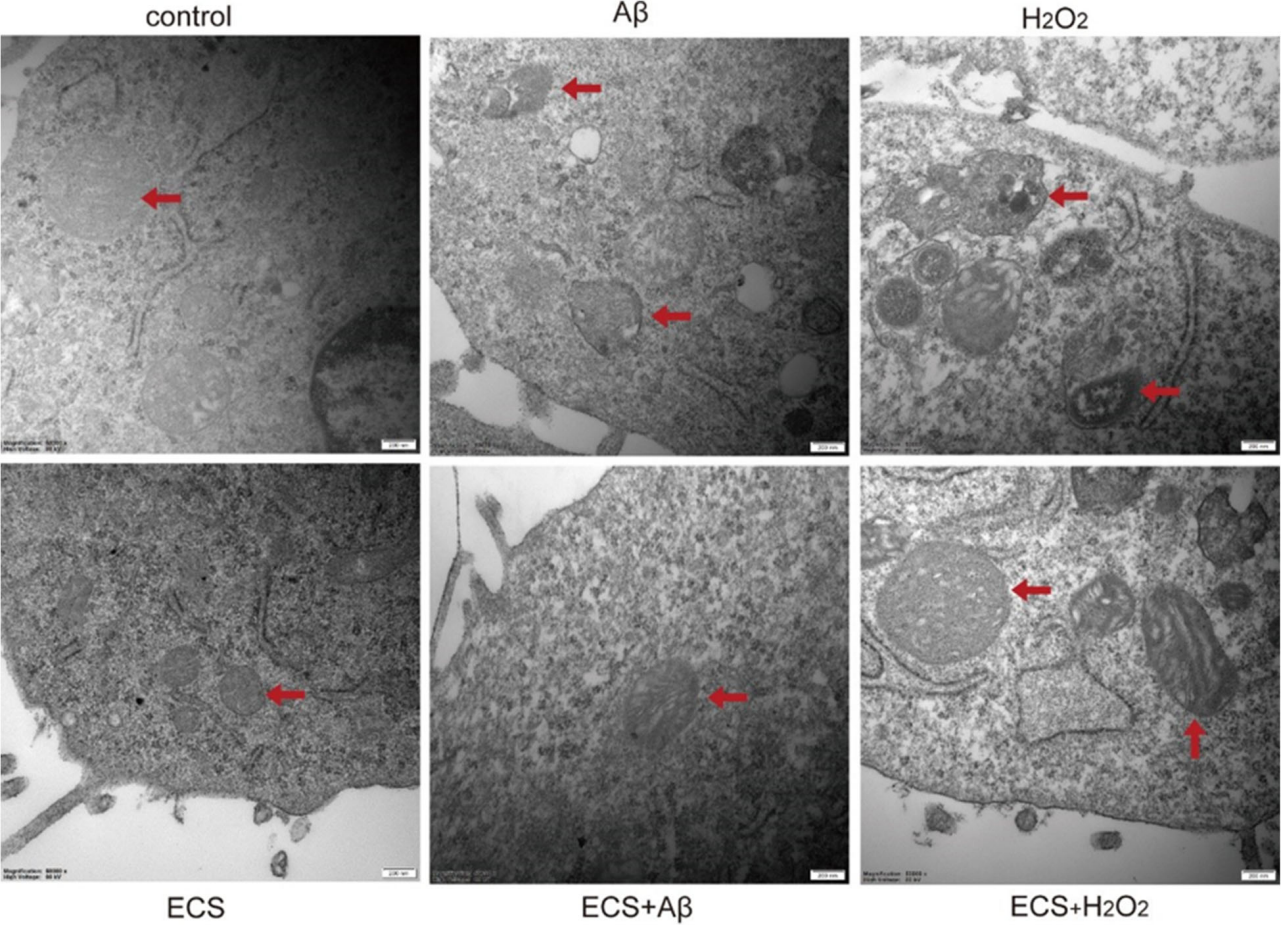
Beneficial effects of ECS on the ultrastructure of cellular mitochondria in the Aβ-induced model and the H_2_O_2_-induced model. Transmission electron microscopy (TEM) was used to examine thin slices or sections of cells in different groups. Representative mitochondria in each group are marked with red arrows. Magnification: 50000x, scale bars: 200 nm

We examined mitochondrial ultrastructures after treatment in Aβ- and H_2_O_2_-exposed cells using TEM. As shown in Fig. 3, the mitochondria in the control and ECS-treated groups exhibited double membranes and neatly arranged lamellar mitochondrial crests. However, in the Aβ- and H_2_O_2_-exposed groups, the mitochondria accumulated damaged and divided fragments and even autophagosomes. ECS ameliorated the damage to the mitochondrial ultrastructure in Aβ- and H_2_O_2_-exposed cells. After treatment, the autophagosomes disappeared, and mitochondrial cristae were clearly observed in these two models.

### Improvements in intracellular ATP levels

**Fig. 4:**
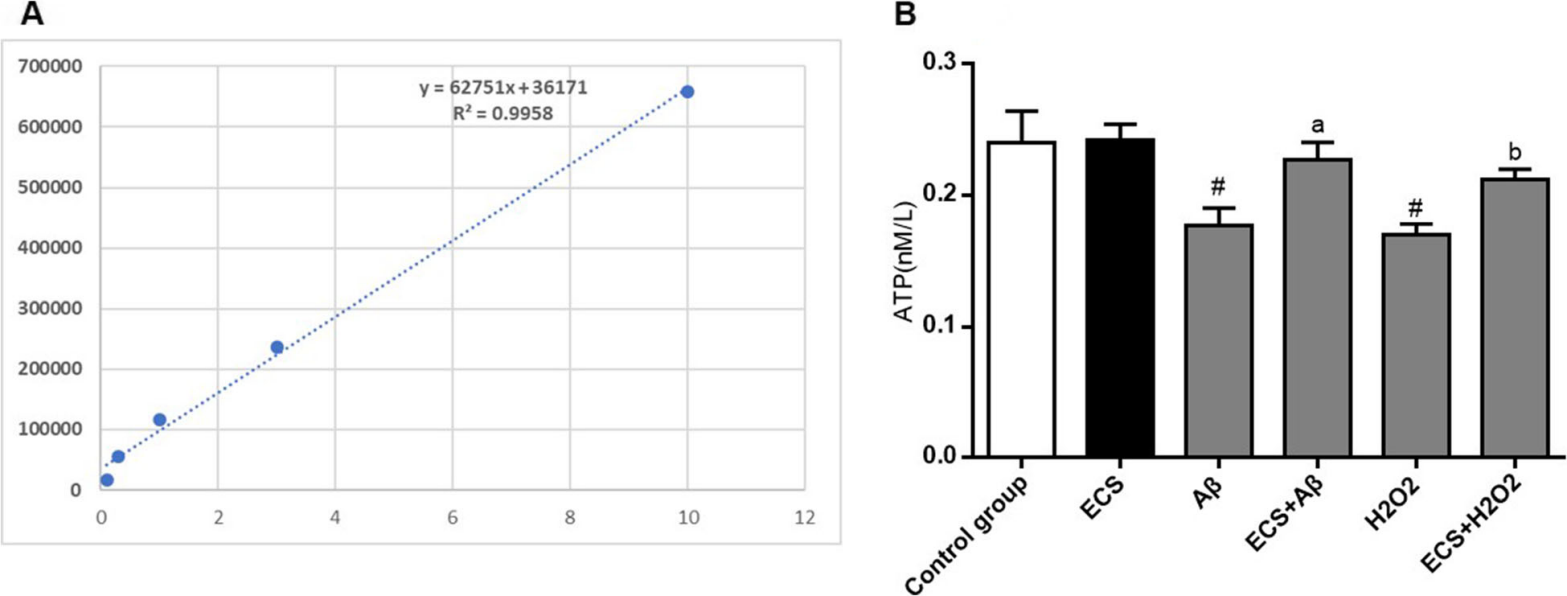
Beneficial effects of ECS on the intracellular ATP levels in the Aβ-induced model and the H_2_O_2_-induced model. **A** Standard curve between the concentration of ATP standard and its absorbance. *R*^*2*^ = 0.9958, indicating good linearity. **B** Changes in intracellular ATP among the six groups. All values are shown as the means ± SEMs from three independent studies. Statistical analyses were performed using one-way ANOVA and Tukey's HSD post hoc comparisons. ^#^*p* < 0.05, vs. control group; ^a^*P* < 0.05, vs. Aβ model group; ^b^*P* < 0.05, vs. H_2_O_2_ model

The calibration curve between the concentration of the ATP standard and its absorbance is shown in Fig. 4A and showed good linearity (*R*^*2*^ = 0.9958). In the Aβ- and H_2_O_2_-exposed groups, the concentrations of ATP were 0.176 ± 0.014 μM and 0.170 ± 0.008 μM, respectively, indicating that Aβ and H_2_O_2_ can cause significant decreases in ATP levels in normal HT22 cells. However, there was no significant difference between the Aβ- and H_2_O_2_-exposed cells. After treatment with ECS, the concentrations of intracellular ATP significantly increased in the two models and were even at the same levels as those in the normal cells.

### Regulatory effects of ECS on ROS

**Fig. 5:**
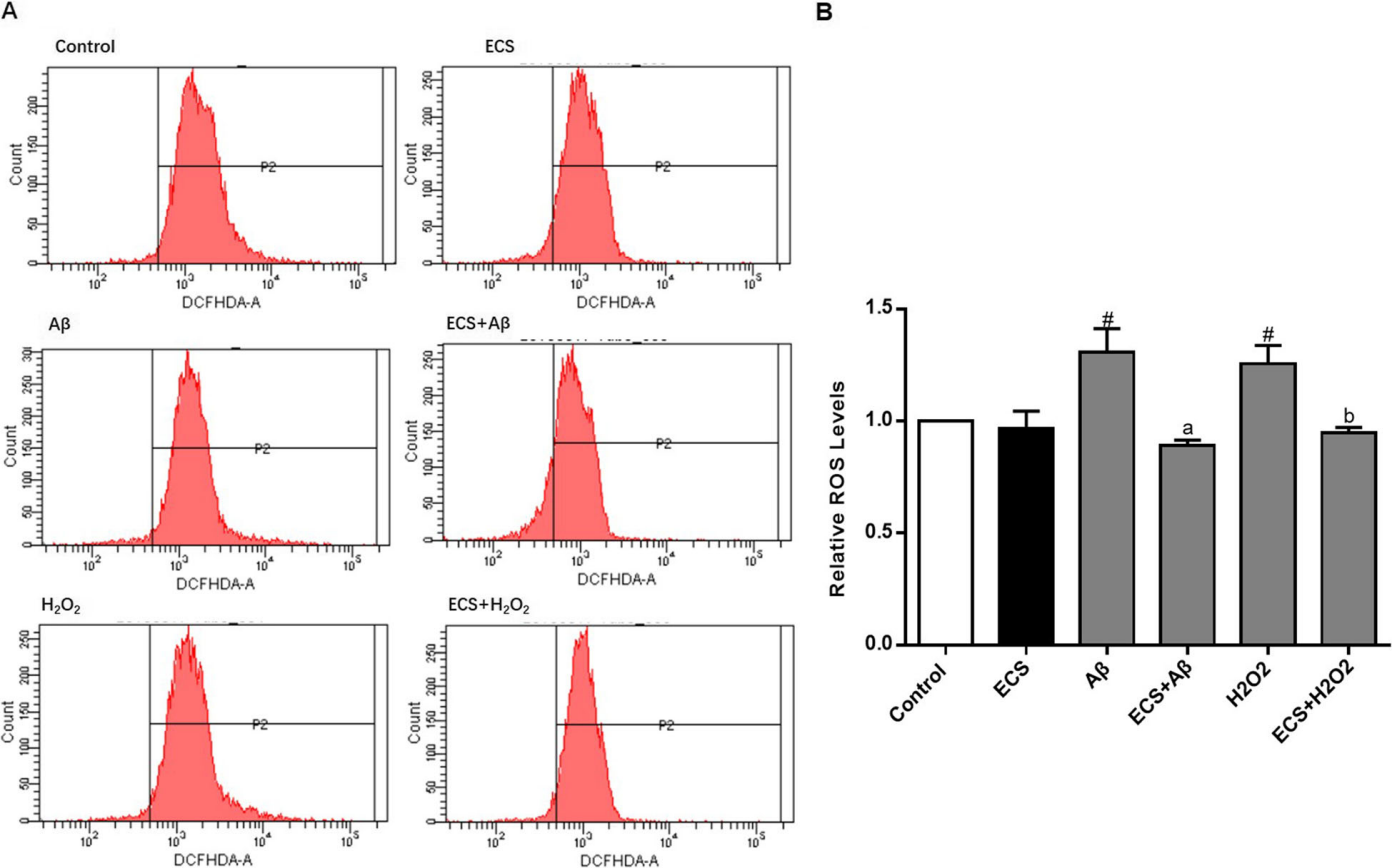
The regulation of intracellular ROS by ECS in the Aβ-induced model and H_2_O_2_-induced model. **A** The results of flow cytometry. **B** The relative level of intracellular ROS in each group compared with the control group. All values are shown as the mean ± SEM from three independent studies. Statistical analyses were performed using one-way ANOVA and Tukey's HSD post hoc comparisons. ^#^*p* < 0.05, vs. control group; ^a^*P* < 0.05, vs. Aβ model group; ^b^*P* < 0.05, vs. H_2_O_2_ model

The relative value of ROS in each group is the ratio of the average fluorescence intensity of that group to the fluorescence intensity in the control group (Fig. 5). The results showed that the relative ROS levels in the Aβ- and H_2_O_2_-exposed cells were 1.36 ± 0.11 and 1.25 ± 0.08, respectively, which were significantly higher than those in the normal cells. However, there was no significant difference between Aβ-exposed and H_2_O_2_-exposed cells. The relative ROS levels in the two treated groups were significantly lower than those in the two model groups. ECS did not significantly change the relative value of ROS in the normal cells.

### Beneficial effects of ECS on the MMP

**Fig. 6:**
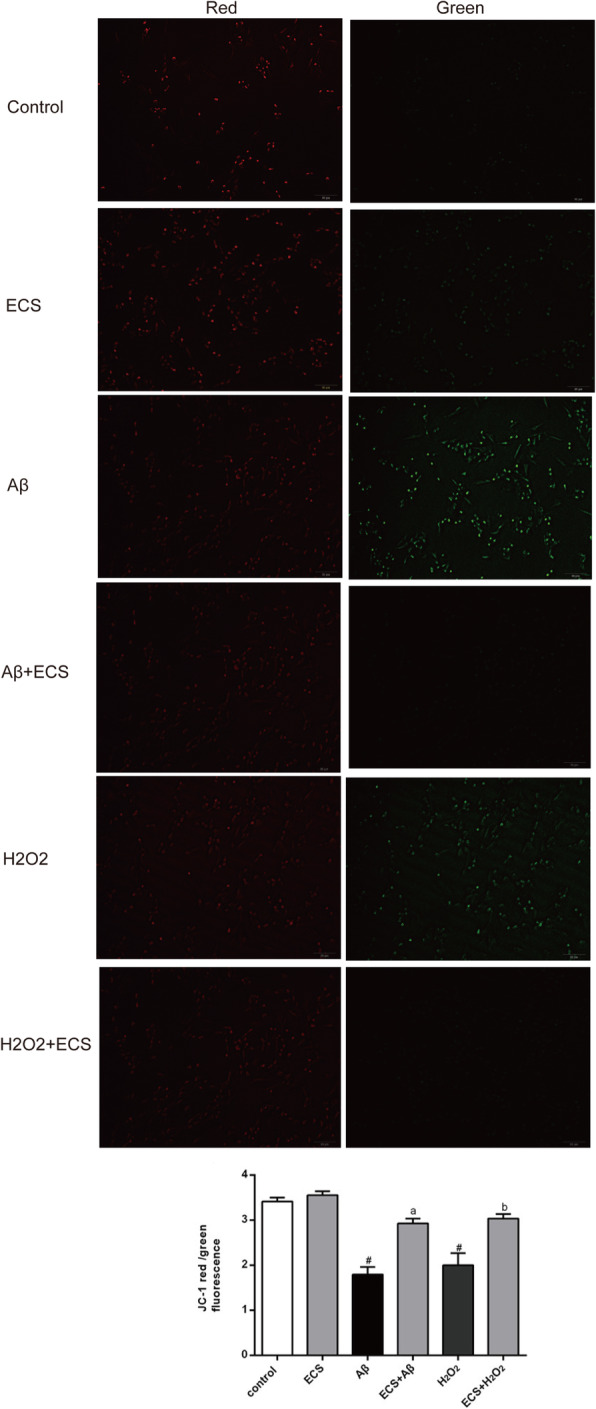
Beneficial effects of ECS on mitochondrial membrane potential in the Aβ-induced model and the H_2_O_2_-induced model. High MMP in cells promotes the formation of dye aggregates that fluoresce red, while low potential promotes monomeric JC-1, which fluoresces green. **A** Mitochondria stained with JC-1 in the different groups. **B** Red/green fluorescence ratios in the different groups. Scale bar: 10 μm. All values are shown as the means ± SEMs from five independent studies. Statistical analyses were performed using one-way ANOVA and Tukey's HSD post hoc comparisons. ^#^*p* < 0.05, vs. control group; ^a^*P* < 0.05, vs. Aβ model group; ^b^*P* < 0.05, vs. H_2_O_2_ model

MMP was observed in HT22 cells by using the mitochondrial membrane fluorescent dye JC-1. Green fluorescence was emitted when the MMP was low, while red fluorescence was emitted when the MMP was high. The red and green fluorescent signals are presented in Fig. 6A. The red/green fluorescent signal ratios in Aβ- and H_2_O_2_-exposed cells were significantly lower than those in the control cells, indicating significant decreases in MMP in these two models (^#^*P* < 0.05). ECS improved MMP in the two model groups (^a^*P* < 0.05; ^b^*P* < 0.05) (Fig. 6B).

### Regulatory effects of ECS on mitochondrial dynamics-related proteins

**Fig. 7:**
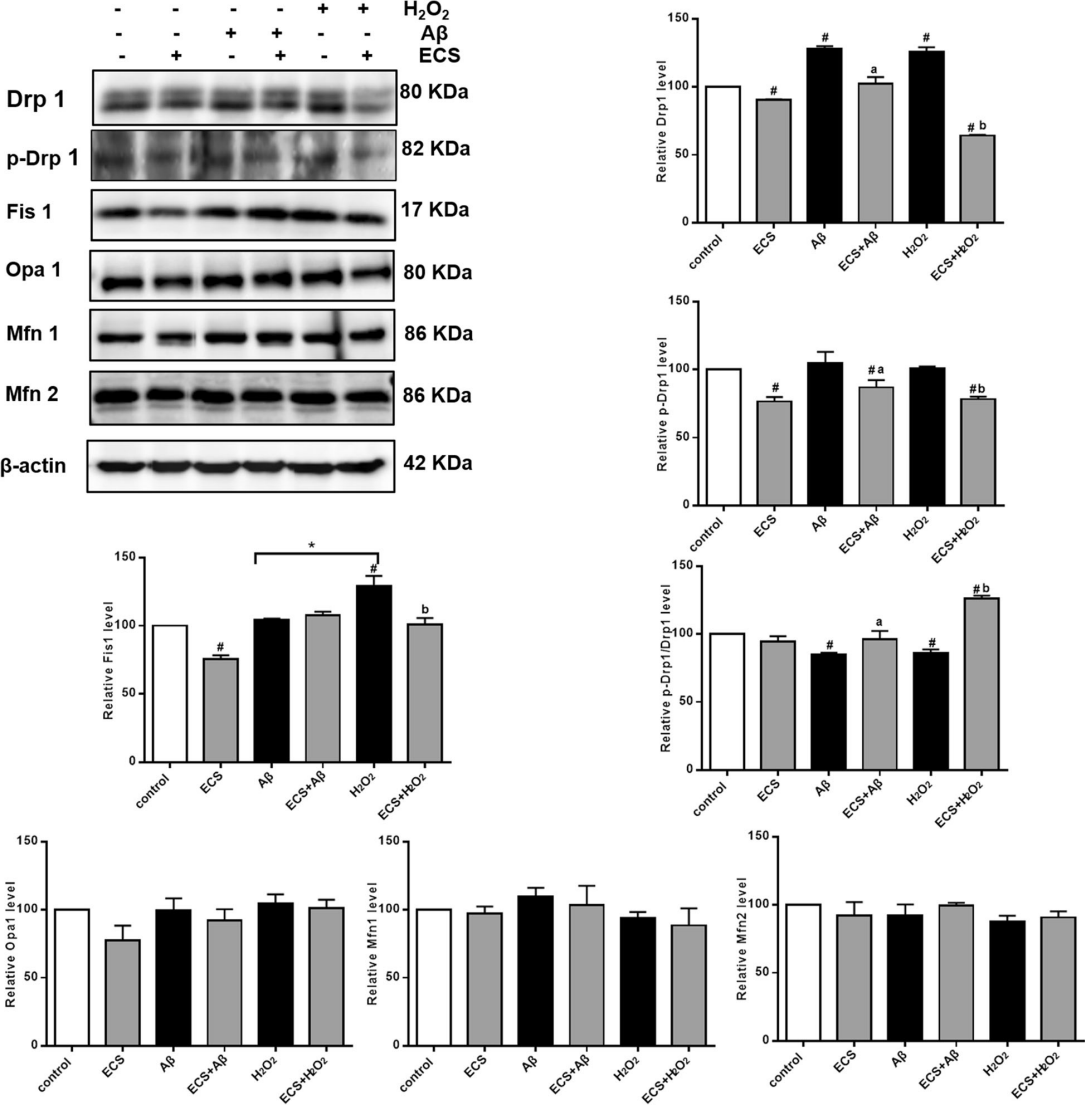
Effects of ECS on the expression of mitochondrial dynamics-related proteins in the Aβ-induced model and the H_2_O_2_-induced model. The expression levels of the proteins in each group are expressed relative to those in the control group. All values are shown as the means ± SEMs from three independent studies. Statistical analyses were performed using one-way ANOVA and Tukey-Kramer post hoc comparisons. ^#^*p* < 0.05, vs. control group; ^a^*P* < 0.05, vs. Aβ model group; ^b^*P* < 0.05, vs. H_2_O_2_ model

The results showed that the expression of Drp1 in the ECS group was significantly lower than that in the control group (^#^*P* < 0.05) (Fig. 7). The expression levels of Drp1 in the Aβ- and H_2_O_2_-exposed models were significantly higher than that in the control group (^#^*P* < 0.05), but there was no significant difference between the two model groups. ECS significantly decreased the expression of Drp1 in Aβ- and H_2_O_2_-exposed cells (^a^*P* < 0.05; ^b^*P* < 0.05). With regard to the expression level of p-Drp1, there were no significant differences in Aβ- and H_2_O_2_-exposed cells compared with normal cells, but ECS significantly decreased the expression of p-Drp1 in both the control group and the two model groups (^#^*P* < 0.05; ^a^*P* < 0.05; ^b^*P* < 0.05). In the Aβ and H_2_O_2_ models, the p-Drp1/Drp1 ratios were significantly lower than that in the control group (^#^*P* < 0.05), and ECS treatment significantly increased the p-Drp1/Drp1 ratios in the two model groups (^a^*P* < 0.05, ^b^*P* < 0.05). Another interesting result was that ECS treatment increased the p-Drp1/Drp1 ratio in the H_2_O_2_ model (^#^*P* < 0.05).

ECS also inhibited excessive mitochondrial fission in H_2_O_2_-exposed cells. The results showed that the expression levels of Fis1 in H_2_O_2_-exposed cells were significantly higher than those in normal cells (^#^*P* < 0.05), while the levels in Aβ-exposed cells were not significantly different. ECS significantly decreased the expression levels of Fis1 in both normal cells and H_2_O_2_-exposed cells (^#^*P* < 0.05, ^b^*P* < 0.05). Interestingly, the expression levels of Fis1 in H_2_O_2_-exposed cells were higher than those in Aβ-exposed cells; this pattern was quite different from the expression patterns of other mitochondrial dynamics-related proteins.

ECS had no significant regulatory effects on the expression of the mitochondrial fusion proteins Opa1, Mfn1, and Mfn2 but showed a tendency to reduce the expression levels of fusion proteins.

### Mechanisms by which ECS regulates mitochondrial kinetic balance in the AD model

#### Changes in intracellular calcium ion concentrations in the Aβ and H_2_O_2_ models

**Fig. 8:**
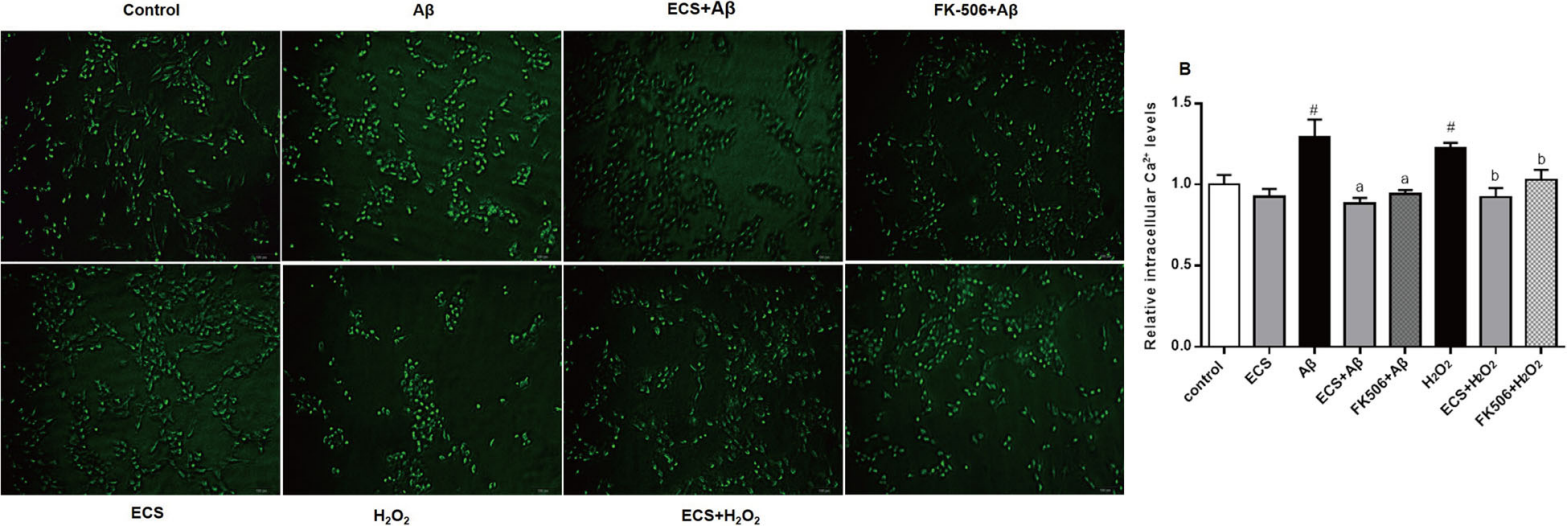
Regulatory effects of ECS on [Ca^2+^]*i* in the Aβ-induced model and the H_2_O_2_-induced model. **A** Fluorescence microscopy images of intracellular calcium ions in the different groups. **B** Concentration of intracellular calcium ions in each group relative to the normal group. All values are shown as the means ± SEMs from three independent studies. Statistical analyses were performed using one-way ANOVA and Tukey's HSD post hoc comparisons. ^#^*p* < 0.05, vs. control group; ^a^*P* < 0.05, vs. Aβ model group; ^b^*P* < 0.05, vs. H_2_O_2_ model

The intracellular calcium ion concentration ([Ca^2+^]*i*) was detected with a fluorescence microscope, and the fluorescence intensity was analyzed using ImageJ software. The [Ca^2+^]*i* in each group was compared to the concentration in the control group. The results showed that the [Ca^2+^]*i* values in Aβ-exposed cells (1.29 ± 0.11) and H_2_O_2_-exposed cells (1.22 ± 0.03) were significantly higher than those in normal cells (^#^*P* < 0.01), but there was no significant difference between the two model groups (Fig. 8). ECS significantly decreased [Ca^2+^]*i* in Aβ-exposed cells and H_2_O_2_-exposed cells to 0.88 ± 0.03 (^a^*P* < 0.01) and 0.92 ± 0.05 (^b^*P* < 0.01), respectively. Similarly, FK506, an inhibitor of calcium ion channels, also significantly decreased [Ca^2+^]*i* in the Aβ-exposed cells and H_2_O_2_-exposed cells (0.94 ± 0.02, ^a^*P* < 0.01; and 1.03 ± 0.61, ^b^*P* < 0.05, respectively), suggesting that ECS and FK506 exert similar pharmacological effects, reducing intracellular calcium ion concentrations.

#### Effects of ECS and FK-506 on the expression of mitochondrial kinetics-related proteins and calcineurin in Aβ and H_2_O_2_ models

**Fig. 9:**
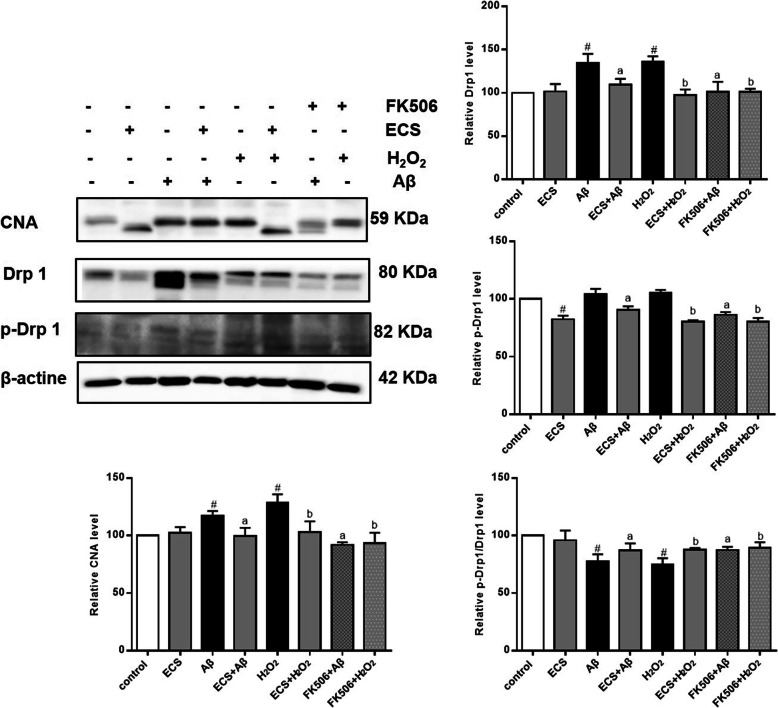
Effects of ECS and FK-506 on the expression of mitochondrial dynamics-related proteins and CaN in the Aβ-induced model and the H_2_O_2_-induced model. The expression levels of the proteins in each group are expressed relative to those in the control group. All values are shown as the means ± SEMs from three independent studies. Statistical analyses were performed using one-way ANOVA and Tukey's HSD post hoc comparisons. ^#^*p* < 0.05, vs. control group; ^a^*P* < 0.05, vs. Aβ model group; ^b^*P* < 0.05, vs. H_2_O_2_ model

To evaluate the effects of intracellular calcium ions on mitochondrial dynamics in the AD models, we detected the expression level of CaN, the intracellular calcium ion concentration, and the phosphorylation of Drp1 (Ser637) after FK506 intervention (Fig. 9). The expression levels of CaN were significantly higher in the Aβ- and H_2_O_2_-exposed cells than in the normal cells (^#^*P* < 0.05), and the CaN expression levels in the H_2_O_2_-exposed cells were slightly higher than those in the Aβ-exposed cells, although the difference was not significant. Additionally, administration of ECS significantly decreased the expression levels of CaN in the two model groups (^a^*P* < 0.05, ^b^*P* < 0.05), and administration of FK-506, which had effects similar to those of ECS, significantly reduced the expression of CaN in the Aβ- and H_2_O_2_-exposed cells (^a^*P* < 0.05, ^b^*P* < 0.05).

FK-506 also significantly decreased the expression levels of Drp1 and p-Drp1 in Aβ- and H_2_O_2_-exposed cells (^a^*P* < 0.05, ^b^*P* < 0.05), similar to ECS. In addition, FK-506 significantly decreased the p-Drp1/Drp1 ratios in Aβ- and H_2_O_2_-exposed cells (^a^*P* < 0.05, ^b^*P* < 0.05), indicating that both ECS and FK-506 can significantly reduce the phosphorylation of Drp1 at serine 637.

## Discussion

HT22 is a subline cloned from the HT4 line of immortalized mouse hippocampal neural precursor cells. It is a valuable cell model for studies on neurodegenerative diseases, such as Alzheimer’s disease [35, 36]. Thus, HT22 cells were used to establish AD models in vitro in this study.

Aβ can induce an AD model because it exerts toxic effects on neuronal cells [37]. Studies have shown that accumulated Aβ on the mitochondrial membrane interacts with mitochondrial matrix components, leading to the degeneration of neuronal synapses [10]. Several studies have shown that Aβ causes intracellular accumulation of ROS and ultimately leads to DNA damage [38]. Meanwhile, ROS cause oxidative stress when their production is higher than their elimination [39]. Therefore, oxidative stress induced by ROS may play a crucial role in the pathogenesis of AD and underlie the mechanism of Aβ-induced neurotoxicity. As ROS and H_2_O_2_ cause oxidative stress, targeting oxidative stress can be considered a therapeutic strategy to ameliorate neurotoxicity in AD.

The mitochondrion is a cellular organelle with a characteristic structure separated from the cytoplasm by the outer and inner mitochondrial membranes. The inner membrane forms invaginations called cristae, which are the main sites of bioenergy transformation in eukaryotes [40]. Early studies demonstrated fragmentation of the mitochondrial network in the AD brain that could cause mitochondrial bioenergetic deficits through mechanisms such as enhancement of ROS generation [41, 42]. This study found that hyperfusion of damaged mitochondria or fragmentation of mitochondria induced by fission occurred in Aβ- and H_2_O_2_-exposed cells. After being treated with ECS, the cells exhibited normal-appearing mitochondria. The lamellar mitochondrial cristae were clearly and neatly arranged, indicating that ECS ameliorated the damage to the mitochondrial ultrastructure.

Mitochondrial morphological alterations are often accompanied by mitochondrial dysfunction [43]. Some studies have suggested that Aβ and H_2_O_2_ both cause mitochondrial dysfunction, excessive generation of ROS, and increases in oxidative stress, which lead to disruption of [Ca^2+^]*i* homeostasis in cells, MPTP opening, and decreases in MMP [10, 13, 18]. In this study, ROS production was increased and ATP levels and MMP were decreased in Aβ- and H_2_O_2_-exposed cells. ECS significantly reduced the intracellular ROS levels to the same levels as in normal cells. Additionally, ECS significantly improved intracellular ATP levels and MMP in Aβ- and H2O2-exposed cells.

Mitochondria are highly dynamic organelles that undergo continuous fusion and fission [44]. Aβ affects the expression levels of these mitochondrial fusion/fission-related proteins by increasing mitochondrial fission gene expression and reducing mitochondrial fusion gene expression [44]. Defects in fission proteins and fusion proteins severely alter mitochondrial morphology and impair mitochondrial function [45]. The mitochondrial fusion proteins Mfn1 and Mfn2 are located in the mitochondrial outer membrane and mainly mediate mitochondrial outer membrane fusion. Mfn2 also plays a crucial role in the conduction of calcium signals [46, 47]. Opa1 is located in the mitochondrial inner membrane and mainly mediates mitochondrial inner membrane fusion, and it also helps maintain the morphology of the mitochondrial cristae [48]. Drp1 and Fis1 are involved in mitochondrial division [47]. Phosphorylation of Drp1 leads to excessive mitochondrial division, which leads to an imbalance in mitochondrial dynamics [21]. In our study, the p-Drp1 (Ser637) expression level in H_2_O_2_-exposed cells showed an increasing trend relative to that in normal cells, but the difference was not significant. ECS intervention significantly decreased the expression of p-Drp1 (Ser637) in both model groups. The p-Drp1/Drp1 ratios in the model groups were decreased significantly, indicating that the phosphorylation levels of Drp1 were increased in the Aβ- and H_2_O_2_-exposed cells. Furthermore, we also found that the expression levels of both Drp1 and p-Drp1 were decreased in the control group treated with ECS, suggesting that the therapeutic targets of ECS may be the receptor molecules of DRP1.

Fis1 is a membrane receptor for Drp1 and is mainly involved in mitochondrial division [22]. Studies have shown that the overexpression of Fis1 and p-Drp1 in mitochondria can inhibit the division and proliferation of mitochondria [22]. Our study results showed that the expression levels of Fis1 were increased significantly in H_2_O_2_-exposed cells compared with normal cells and were also higher than those in Aβ-exposed cells. ECS significantly reduced the expression levels of Fis1 in H_2_O_2_-exposed cells. However, the expression levels of Fis1 in Aβ-exposed cells showed a slight increasing trend without a significant difference, similar to the results in another study [49]. Overall, in this study, only the expression levels of Drp1, p-Drp1, and Fis1 changed significantly. The expression of other proteins related to mitochondrial fusion, namely, Opa1, Mfn1, and Mfn2, showed no significant differences. This was contrary to our expectations but was similar to the results of other studies [49, 50]. These results suggest that the phosphorylation of Drp1 may be an essential factor triggering mitochondrial division and indirectly affect the expression levels of other mitochondrial kinetics-related proteins [51].

Calcium signals affect mitochondrial dynamics by activating calcium-sensitive effectors, such as calcineurin (CaN) [21]. Calcineurin is a serine/threonine protein phosphatase (PPase) with a critical role in signal transduction; for example, it regulates the dephosphorylation of Drp1 at Ser337 [52, 53]. Our results showed that ECS reduced the concentrations of Ca^2+^ and the expression levels of CaN in Aβ- and H_2_O_2_-exposed cells and decreased Drp1 phosphorylation. Thus, ECS can maintain mitochondrial ultrastructure and function in AD models by reducing intracellular calcium ion concentrations, decreasing the expression level of CaN, promoting Drp1 dephosphorylation, and maintaining the balance of mitochondrial dynamics.

There were some limitations in this study. First, the study investigating the effect of ECS on mitochondrial dysfunction was a preliminary in vitro study. Behavioral and pharmacological studies in animals should be considered in the future. Second, the components of ECS that play major roles in the therapeutic effects remain unclear and need further study.

## Conclusion

According to our study results, ECS enhanced MMP, increased intracellular ATP levels, protected mitochondrial ultrastructure and function, and decreased intracellular ROS levels in the Aβ- and H_2_O_2_-induced AD models in vitro. Additionally, ECS regulated the expression levels of mitochondrial dynamics-related proteins; for example, it reduced the dephosphorylation of Drp1 at Ser637 and reduced the expression of Fis1 in the H_2_O_2_-induced model. Further study was performed to explore the mechanism by which ECS reduced the phosphorylation of Drp1 at Ser637. The results indicated that ECS reduced overload of intracellular calcium ions, decreased the expression levels of CaN, and increased the dephosphorylation of Drp1 in Aβ- and H_2_O_2_-exposed cells. Therefore, ECS can maintain the balance of mitochondrial dynamics by reducing overload of intracellular calcium ions and increasing dephosphorylation of Drp1 at Ser637, thereby increasing MMP and intracellular ATP levels and maintaining the stability of mitochondrial structure and function in Aβ- and H_2_O_2_-induced AD models. Collectively, our findings provide evidence regarding mitochondrial status and successive changes in AD and suggest a new therapeutic strategy for AD patients.

## Supplementary Information

**Additional file 1 Fig. S1.** Effects of ECS on cell viability of HT22 under different action times and concentrations. Cells were treated with 12.5 μg/mL, 25 μg/mL, 50 μg/mL, 100 μg/mL, 200 μg/mL, 400 μg/mL, and 800 μg/mL of ECS, respectively. CCK-8 was added after the cells were treated with ECS for 12 and 24 h, and the cell viability was calculated according to the OD value. *P<0.05, compared with the control group (*n* = 6). **Fig. S2.** Effects of Aβ on the viability of HT22 cells. Different concentration of Aβ, 10 μM, 20 μM, 40 μM, 80 μM, respectively acted on the HT22 cells for 12 h and 24 h. The cell ability under different concentration and administration time of Aβ was calculated according to the OD value. *P<0.05, compared with control group. **Fig. S3.** Effect of H_2_O_2_ on the viability of HT22 cells. Different concentration of H_2_O_2_, 50 μM, 100 μM, 200 μM, 400 μM, 800 μM, respectively acting on HT22 cells for 2 h, 4 h, 8 h, 12 h, and 24 h. The cell ability under different concentration and different administration time of H_2_O_2_ was calculated by the OD value. *P<0.05, compared with control group.

## Data Availability

All data generated or analysed during this study are included in this published article and its ikmentary information files.

## Abbreviations

AD: Alzheimer’s disease
ECS: The extracts of *Cynomorium songaricum*
MMP: Mitochondrial membrane potential
ROS: Reactive oxygen species
SP: Senile plaques
NFT: Neurofibrillary tangle
APP: Amyloid precursor protein
mtDNA: Mitochondrial DNA
MPTP: Mitochondrial permeability transition pores
Drp1: Dynamin-related protein 1
Fis1: Fission 1
Mfn1: Mitofusin l
Opa 1: Optic atrophy 1
LOD: Limit of detection
LOQ: Limit of quantitation
DMEM: Dulbecco’s modified eagle media
FBS: Fetal bovine serum
H_2_O_2_: Hydrogen peroxide
CCK-8: Cell Counting Kit-8
PBS: Phosphate-buffered saline
TEM: Transmission electron microscope
SDS-PAGE: Sodium dodecyl sulfate-polyacrylamide gel electrophoresis
PVDF: Polyvinylidene difluoride
TBST: Tris Buffered Saline with Tween 20
DPBS: Dulbecco’s phosphate-buffered saline
HPLC: High-performance liquid chromatography
RSD: Relative standard deviation
CaN: Calcineurin
