# Supplementary material for: Extract of Cynomorium songaricum ameliorates mitochondrial ultrastructure impairments and dysfunction in two different in vitro models of Alzheimer’s disease
Source: BMC Complement Med Ther. 2021 Aug 9;21:206. doi: 10.1186/s12906-021-03375-2 (PMC8351341; doi:10.1186/s12906-021-03375-2)
Supplement: Supplementary file 1 — Additional file 1 Fig. S1. Effects of ECS on cell viability of HT22 under different action times and concentrations. Cells were treated with 12.5 μg/mL, 25 μg/mL, 50 μg/mL, 100 μg/mL, 200 μg/mL, 400 μg/mL, and 800 μg/mL of ECS, respectively. CCK-8 was added after the cells were treated with ECS for 12 and 24 h, and the cell viability was calculated according to the OD value. *P<0.05, compared with the control group (n = 6). Fig. S2. Effects of Aβ on the viability of HT22 cells. Different concentration of Aβ, 10 μM, 20 μM, 40 μM, 80 μM, respectively acted on the HT22 cells for 12 h and 24 h. The cell ability under different concentration and administration time of Aβ was calculated according to the OD value. *P<0.05, compared with control group. Fig. S3. Effect of H2O2 on the viability of HT22 cells. Different concentration of H2O2, 50 μM, 100 μM, 200 μM, 400 μM, 800 μM, respectively acting on HT22 cells for 2 h, 4 h, 8 h, 12 h, and 24 h. The cell ability under different concentration and different administration time of H2O2 was calculated by the OD value. *P<0.05, compared with control group. [file 12906_2021_3375_MOESM1_ESM.docx]

Supplementary Material

1.Effects of ECS on cell viability

Cells were seeded in 96-well plates at a density of 8×10^4^ cell/mL, 100 μL for each well. After 24 hours, cells were treated with 12.5 μg/mL, 25 μg/mL, 50 μg/mL, 100 μg/mL, 200 μg/mL, 400 μg/mL, and 800 μg/mL of ECS , respectively. CCK-8 was added after the cells were treated with ECS for 12 and 24 hours, and then the OD value was detected at 450 nm. The cell viability was calculated according to the OD value. According to the experimental results (Fig. S1), when the concentration of ECS was 400μg/mL and 800 μg/mL, the cell ability in the treatment group was lower than that in the control group no matter whether the administration time of ECS was 12h or 24h, indicating that 400 μg/mL and 800μg/mL of ECS may inhibit the cell ability of HT22 cells. When the concentration of ECS was 200 g/mL and the time of ECS administration was 24 hours, the cell ability of HT22 cells was the highest, so the treatment concentration of ECS was determined to be 200 μg/mL in the subsequent experiments.

2. Establishment of two in vitro cell models of AD

2.1 Aβ-induced HT22 cellsss

The cell ability under different concentrations and administration time of Aβ was calculated according to the OD value. Compared with the same concentration of Aβ, the cell ability in the 24-hour Aβ group was lower than 12-hour Aβ group, suggesting that with the action time of Aβ extending, the greater the damage to HT22 cells caused (Figure S2). While under the same action time, the cell ability of HT22 cells decreased with the increase of the concentration of Aβ, which means the greater damage to the cells was caused. According to the results of cell ability of HT22 cells at different action times and different concentrations, 20 μM of Aβ was determined to act on the HT22 cells for 24 hours in the subsequent experiments to mimic the AD model, with the cell ability was 69.62±1.33% (Fig. S2).

2.2 H_2_O_2_-induced HT22 cells.

The cell ability under different concentrations and different administration times of H_2_O_2_ was calculated by the OD value. According to the results under the same administration time of H_2_O_2_, the cell ability of HT22 cells decreased with the increase of the H_2_O_2_ concentration; while under the same concentration of H_2_O_2_, the cell ability of HT22 cells decreased with the increase of the administration time. When the concentration of H_2_O_2_ was higher than 200 μM, that was, the concentration of H_2_O_2_  in the experiment was 200 μM, 400 μM, 800 μM, the cell ability was significantly lower than that when the concentration of H_2_O_2_ was 50 μM and 100 μM. However, when the concentration of H_2_O_2_ was higher than 200 μM, there was no significant difference in cell ability at the same administration time although the different concentrations of H_2_O_2_. The 200 μM of H_2_O_2_ with 2 hours administration time was determined to act on HT22 cells for two hours, with the cell ability was 66.90±3.24%, to establish the invitro AD model (figure S3).

**Fig. S1.** Effects of ECS on cell viability of HT22 under different action times and concentrations. Cells were treated with 12.5 μg/mL, 25 μg/mL, 50 μg/mL, 100 μg/mL, 200 μg/mL, 400 μg/mL, and 800 μg/mL of ECS , respectively. CCK-8 was added after the cells were treated with ECS for 12 and 24 hours, and the cell viability was calculated according to the OD value. *P＜0.05, compared with the control group (n=6).


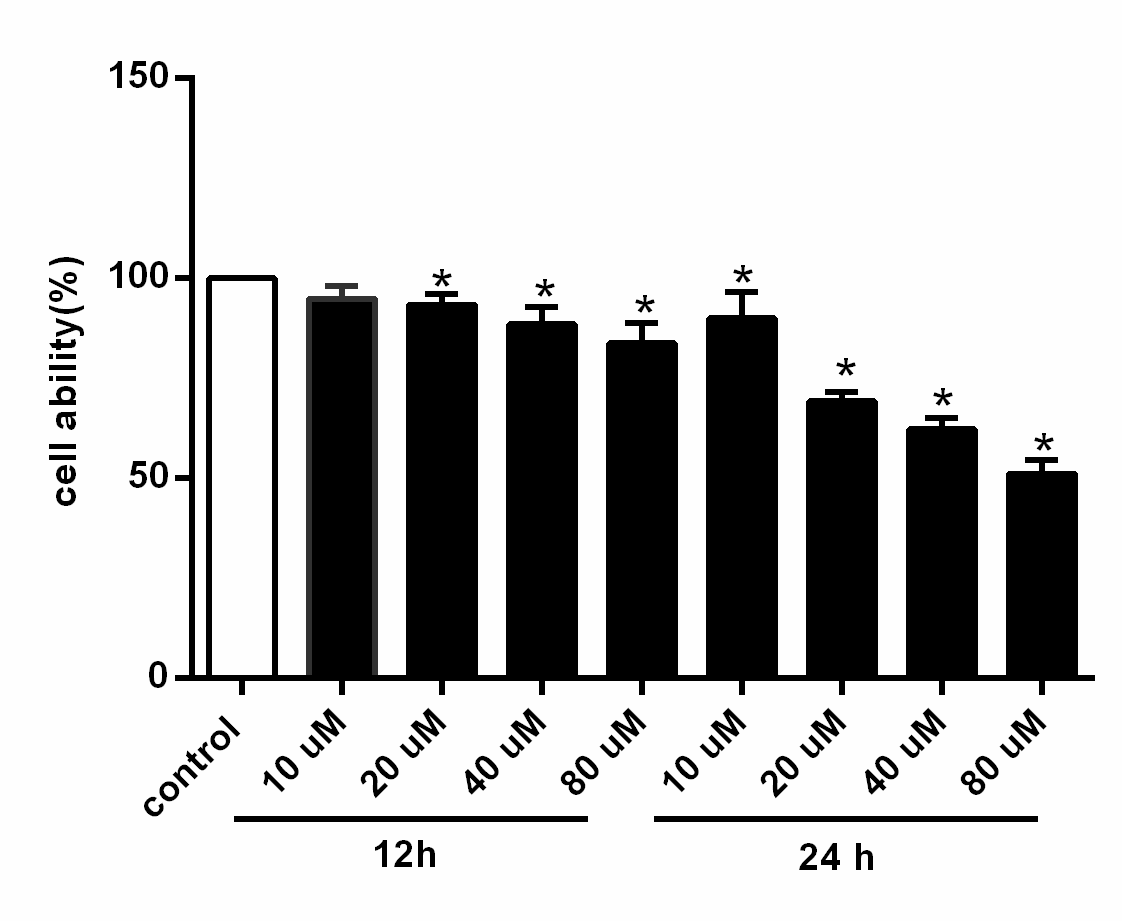


**Fig S2.** Effects of Aβ on the viability of HT22 cells. Different concentration of Aβ, 10 μM, 20μM, 40μM, 80μM, respectively acted on the HT22 cells for 12 hours and 24 hours. The cell ability under different concentration and administration time of Aβ was calculated according to the OD value. *P＜0.05, compared with control group.

**Figur S3.** Effect of H_2_O_2_ on the viability of HT22 cells. Different concentration of H_2_O_2_, 50 μM, 100 μM, 200 μM, 400 μM, 800μM, respectively acting on HT22 cells for 2h, 4h, 8h, 12h, and 24h. The cell ability under different concentration and different administration time of H_2_O_2_ was calculated by the OD value. *P＜0.05，compared with control group.
